# Detection and Pattern Recognition of Chemical Warfare Agents by MOS-Based MEMS Gas Sensor Array

**DOI:** 10.3390/s25082633

**Published:** 2025-04-21

**Authors:** Mengxue Xu, Xiaochun Hu, Hongpeng Zhang, Ting Miao, Lan Ma, Jing Liang, Yuefeng Zhu, Haiyan Zhu, Zhenxing Cheng, Xuhui Sun

**Affiliations:** 1 Institute of NBC Defence, Beijing 102205, China; xmx_tgcfbwjj@163.com (M.X.); hhuuxxcc@163.com (X.H.); hongpeng_zhang@sina.com (H.Z.); 13552291577@163.com (T.M.); malan112@126.com (L.M.); catherin7112@163.com (J.L.); 15313098531@139.com (Y.Z.); zhuhyuse@163.com (H.Z.); 2 Jiangsu Key Laboratory for Carbon-Based Functional Materials & Devices, Institute of Functional Nano & Soft Materials (FUNSOM), Soochow University, 199 Ren’ai Road, Suzhou 215123, China

**Keywords:** CWAs, MEMS, MOS-based sensor array, feature extraction, pattern recognition

## Abstract

**Highlights:**

**What are the main findings?**

- A sensor array consisting of 24 MOS-based MEMS sensors with good sensing performance in relation to chemical warfare agents (CWAs), a simple device structure, small size, and low power consumption was developed.
- Pattern recognition methods, such as principal component analysis and machine learning algorithms, were applied and accurately classified five CWAs based on features extracted from the initial kinetic characteristics and dynamic change characteristics of sensing response in testing data.

**What are the implications of the main findings?**

- The developed sensor array can meet the needs for qualitatively detecting and providing an early warning regarding low concentrations of CWAs.
- The sensor array can be integrated into portable devices and is driven by algorithms to realize the rapid, in situ, and on-site detection and identification of CWAs.

**Abstract:**

Chemical warfare agents (CWAs), including hydrogen cyanide (AC), 2-[fluoro(methyl)phosphoryl]oxypropane (GB), 3-[fluoro(methyl)phosphoryl]oxy-2,2-dimethylbutane (GD), ethyl S-(2-diisopropylaminoethyl) methylphosphonothioate (VX), and di-2-chloroethyl sulfide (HD), pose a great threat to public safety; therefore, it is important to develop sensing technology for CWAs. Herein, a sensor array consisting of 24 metal oxide semiconductor (MOS)-based MEMS sensors with good gas sensing performance, a simple device structure (0.9 mm × 0.9 mm), and low power consumption (<10 mW on average) was developed. The experimental results show that there are always several sensors among the 24 sensors that show good sensing performance in relation to each CWA, such as a relatively significant response, a broad detection range (AC: 5.8–89 ppm; GB: 0.04–0.47 ppm; GD: 0.06–4.7 ppm; VX: 9.978 × 10^−4^–1.101 × 10^−3^; HD: 0.61–4.9 ppm), and a low detection limit that is lower than the immediately dangerous for life and health (IDLH) level of the five CWAs. This indicates that these sensors can meet the needs for qualitative detection and can provide an early warning regarding low concentrations of CWAs. In addition, features were extracted from the initial kinetic characteristics and dynamic change characteristics of the sensing response. Finally, principal component analysis (PCA) and machine learning algorithms were applied for CWA classification. The obtained PCA plots showed significant differences between groups, and the narrow neural network among the machine learning algorithms achieves a prediction accuracy of nearly 100.0%. In summary, the proposed MOS-based MEMS sensor array driven by pattern recognition algorithms can be integrated into portable devices, showing great potential and practical applications in the rapid, in situ, and on-site detection and identification of CWAs.

## 1. Introduction

The human olfactory system can provide odor information to the nervous system, which can help to determine the source and concentration range of odors, thereby providing an early warning regarding possible chemical hazards. However, chemical warfare agents (CWAs) with high toxicity may cause serious harm once they are inhaled or come into contact with the skin, so it is clearly impossible to distinguish the type and concentration of CWAs through humans’ sense of smell. Therefore, it is of great practical significance to develop CWA detection technology and fabricate an electronic nose (e-nose) that can replace the human olfactory and nervous system.

Nowadays, there are a variety of techniques used for the detection of CWAs, and each has its own advantages and disadvantages, as listed in Table S1. Gas chromatography and gas chromatography-mass spectrometry are accurate, sensitive, and reliable, while liquid chromatography has a high separation capacity for complex samples and a strong qualitative ability. However, these methods are time-consuming, bulky, and are usually fixed in a laboratory. Infrared spectroscopy has the advantages of a high sensitivity, a fast response, a low limit of detection (LOD), and no need for sample preparation, but it is complex, has a large size, and incurs a high cost, while ion mobility spectrometry detectors are sensitive and relatively portable, but they have poor selectivity and high false alarm rates and can easily be affected by fluctuations in temperature, pressure, and humidity [1,2]. In recent decades, attention on the development of gas sensors for CWA detection has been growing because sensing technologies have advantages such as easy operation, portability, a low cost, and fast responses [3,4], but the reported studies mostly used simulant agents instead of actual CWAs as target gases because of their strong toxicity, and because CWAs are forbidden [5,6,7,8,9,10,11]. This means that the sensing performance of the developed sensors in relation to actual CWAs in the literature is not clear. Our team and collaborators have made efforts to study gravimetric sensors, such as silicon nanowire sensors [12,13], micro-cantilevers [14], and quartz crystal microbalance sensors [15,16], for the detection of actual CWAs. The resulting sensors have great potential in the detection of hydrocyanic acid (HCN, AC) and the simulant gases of CWAs, i.e., dimethyl methylphosphonate (DMMP), but they still have disadvantages such as a slow response rate and incomplete reversibility [12,13], low sensitivity [14], and a low recovery rate [15,16].

In order to mitigate the shortcomings of existing CWA detection technologies to the greatest extent and to ensure the availability of suitable CWA detection techniques across diverse application scenarios, metal oxide semiconductor (MOS) gas sensors have gradually attracted increasing attention. MOS gas sensors have been widely studied and applied in the field of conventional toxic and harmful gas detection due to their advantages of high sensitivity, good stability, and a simple structure [17,18,19,20,21]. In addition, MOS sensors for the detection of simulant agents of CWAs have also been developed to a certain extent [5,6,7,8,9,10,11,22,23,24,25,26]. However, the development of MOS sensors for the detection of actual CWAs remains a challenge. First of all, due to the high toxicity of CWAs, the appropriate qualifications and security of the laboratory environment, the expertise of operators, and complete protective facilities are strictly required. Secondly, although the simulant gases have similar functional groups or structures to their corresponding CWAs, they still need to be tested in practice as to whether MOS sensors developed with simulants as the detection target can be directly applied to the detection and identification of CWAs. In addition, it has been shown that certain elements such as P, S, Cl, and their functional groups in the simulants may cause a poisoning effect on the sensing material and thereby affect its recovery, stability, working life, etc. [11,23,25]. Unfortunately, CWAs not only contain particular elements such as P, S, Cl, and F, but also have a more complex molecular structure compared with their simulants, which may result in a stronger poisoning effect on MOS materials.

In addition to the negative effects caused by the special properties of CWAs, MOS sensors also have some inherent limitations. Among them, a key problem is that sensors based on a single MOS material often have limited selectivity and are cross-sensitive to multiple gases, making it difficult to identify gases. In response to this, an effective strategy may be to construct sensor arrays and combine them with artificial intelligence (AI) algorithms. However, the MOS sensors developed so far are mostly traditional ceramic tube or chip structures, and the sensor arrays built on them usually have disadvantages such as a large size, high power consumption, and difficulty with regard to integration, limiting their practical application [22,27,28,29]. Therefore, the introduction of the micro-electro-mechanical system (MEMS) [30,31,32,33] is of great practical significance for the development of a miniaturized, intelligent, low-power, portable sensor array to achieve the microscale sensing and accurate identification of a variety of CWAs.

Previously, our team studied the gas sensing performance and pattern recognition ability of an electronic nose (e-nose) based on 24 MOS-based MEMS sensors for five CWA simulants. Our results showed that the sensors in the array exhibited good gas sensing performance and distinguishable characteristics in gas sensing test data. The device also realized the discrimination of five simulants with pattern recognition methods. Additionally, the gas sensing tests in the work spanned several months, and the sensor performance remained relatively stable during the period, indicating remarkable stability and a long working life. This previous work laid a foundation for the study of the detection and identification of actual CWAs using relevant sensors.

In this work, the gas sensing performance of 24 MOS-based MEMS sensors in relation to five kinds of CWAs, i.e., hydrogen cyanide (AC), 2-[fluoro(methyl)phosphoryl]oxypropane (sarin, GB), 3-[fluoro(methyl)phosphoryl]oxy-2,2-dimethylbutane (soman, GD), ethyl S-(2-diisopropylaminoethyl) methylphosphonothioate (VX), di-2-chloroethyl sulfide (mustard gas, HD), was investigated. The chemical structures of these five CWAs are shown in Table S2. To the best of our knowledge, no sensors for VX detection have been reported previously in the literature. Based on the obtained data, the features were extracted to construct datasets. Finally, appropriate pattern recognition methods were applied to classify the CWAs.

## 2. Materials and Methods

### 2.1. Materials and Devices

AC, GB, GD, VX, and HD have a purity higher than 98%. Sodium hydroxide (NaOH, AR) was purchased from Tianjin Fuchen Chemical Reagent Co., Ltd. (Tianjin, China), and dichloromethane (DCM, GR) was purchased from Innochem Co., Ltd. (Beijing, China). NaOH was used to absorb AC in the gas stream for the colorimetric quantification of the AC concentration. DCM was used to absorb GB, GD, VX, and HD for the quantification of their concentration by means of gas chromatography (GC, Agilent 6890 N, Santa Clara, CA, USA).

**Figure 1:**
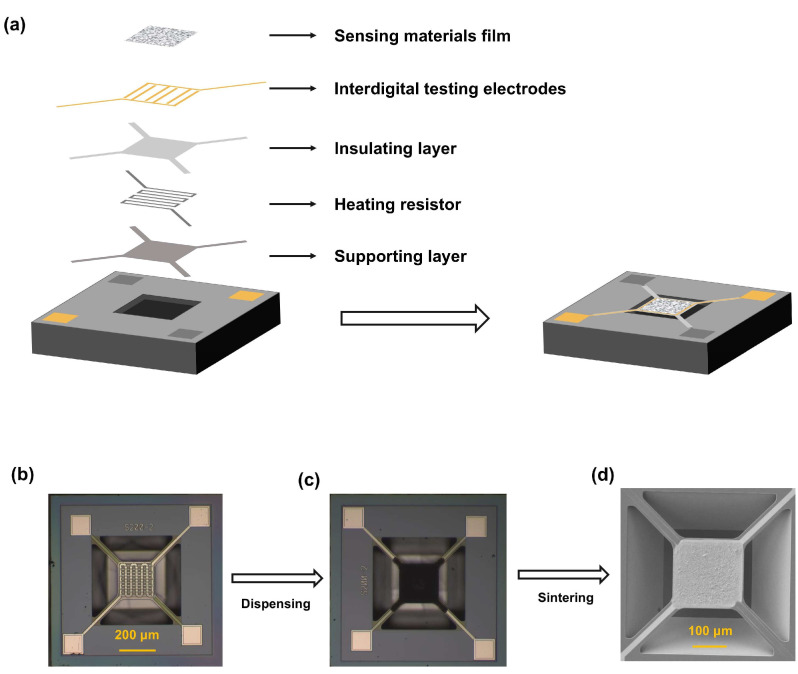
Schematic diagram of a MEMS sensor (**a**) The micro-hot plate structure in the MEMS sensor (**b**) Microscope image of the micro hotplate (**c**) Microscope image of the micro hotplate after dispensing sensing material on it (**d**) SEM images of the sensing film formed after the sintering of the dispensed material.

The sensors recorded as S1–S24 in this work were obtained from Suzhou Huiwen Nanotechnology Co., Ltd. (Suzhou, China). The structure of the silicon-based micro-hot plates (MHPs) in the sensors is shown in Figure 1a; they mainly include a film of sensing materials, interdigital testing electrodes, an insulating layer, a heat resistor, and a supporting layer. The sensing area of each MHP was loaded with sensing materials, which were mainly different forms of modified SnO_2_, ZnO, and WO_3_, etc. (Table S3). All the materials were prepared using the Sol-Gel method. As shown in Figure 1b–d, the materials were dispensed to the sensing area of the MHP and then sintered at a high temperature greater than 350 °C, finally forming a sensing film on the MHP. During the gas sensing test, different heating voltages were applied to the heat resistor of each sensor as listed in Table S3, resulting in a different specified local temperature between 150 °C and 350 °C, providing enough energy for gas sensing on the MOS’s surface. The sensors also had a small size (0.9 mm × 0.9 mm), low power consumption (<10 mW on average), and could easily be integrated. All of the abovementioned advantages were the basis for choosing these sensors.

### 2.2. Gas Sensing Tests

**Figure 2:**
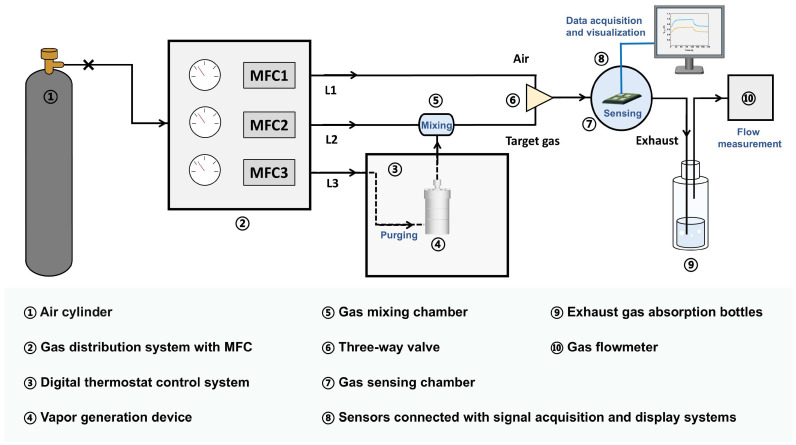
Schematic diagram of the dynamic gas distribution system for CWA sensing tests.

Figure 2 shows a schematic diagram of the dynamic gas distribution system for CWA sensing tests. Air was used as the carrier gas, and three types of air were separated and recorded as L1, L2, and L3, where L1 is the clean air used for sensor aging and recovery, L2 is the clean air introduced into the gas mixing chamber, and L3 is the flow that was purged through the CWA vapor generation device which ultimately contained a certain amount of CWAs. L3 was also introduced into the gas mixing chamber and diluted by L2. By switching the three-way valve, clean air or a mixture of L2 and L3 that contained CWAs was finally introduced into the gas sensing chamber where the sensor array was placed. Due to the buffering of the gas mixing chamber and the length of the gas path, the concentration of the CWAs in the mixture to be introduced into the gas sensing chamber was relatively uniform, and the flow was stable.

At the same time as the gas sensing test, the gas discharged from the gas sensing chamber flowed through the absorption bottle, and the CWAs in it were absorbed by the appropriate absorption solutions. Subsequently, the concentration of CWAs was analyzed by means of pretreatment and quantitative detection methods such as colorimetry or gas chromatography.

According to the immediately dangerous for life and health (IDLH) level of CWAs, the concentration range of the CWA flow during the gas sensing tests was controlled based on the temperature range (−20–40 °C) of the thermostat control system and the adjustable flow range of the gas distribution system. The final concentration ranges of the five CWAs to which the sensors were exposed were as follows: AC: 5.8–89 ppm; GB: 0.04–0.47 ppm; GD: 0.06–4.7 ppm; VX: 9.978 × 10^−4^–1.101 × 10^−3^; HD: 0.61–4.9 ppm. It should be noted that the minimum detection concentrations of GD and HD are higher than their IDLH level, as shown in Table S4. This is due to the failure in the quantitative detection of very low concentrations of GD and HD caused by the limited detection ability of GC. In order to determine the relationship between the gas sensing performance of the sensors and the concentration of CWAs, gas sensing tests were carried out in a measurable concentration range, and the limit of detection (LOD) was calculated according to the theoretical formula LOD = 3 N/M, where N represents the baseline noise and M represents the slope of the response–concentration curve at low concentrations.

The details of the testing processes are as follows. Firstly, the gas mixture containing CWAs was introduced into the gas sensing chamber for 5 min to induce a reaction from the sensors. Then, clean air (L1) was introduced for at least 10 min to allow the sensors to recover. The above processes were repeated to perform cyclic tests, and the output signals were recorded at a frequency of 1 Hz. Overall, 7–10 concentrations of each CWA were tested, and each concentration was tested 5–8 times. During the test, the room temperature was kept at 15–25 °C, the relative humidity of the environment varied within the range of 10–80%, and the relative humidity of the carrier gas was maintained at 2–5%.

The dynamic response (R), the steady-state response (SR), and the maximum response (MR) during gas sensing were calculated as V_g_/V_a_, V_s_/V_a_, and V_m_/V_a_, respectively. Here, V_a_ is the stable baseline voltage signal obtained when sensors are aged in air, V_g_ is the real-time voltage signal during the gas sensing process, V_s_ is the signal when V_g_ tends to stabilize, and V_m_ is the maximum value of V_g_ in the response stage. The response time (t_res_) and recovery time (t_rec_) are defined as the time needed for the output signal of the sensors to change by 90% after exposure to the target gases and air, respectively.

## 3. Results and Discussion

### 3.1. Gas Sensing Performances

#### 3.1.1. Performance of the Sensors in Relation to AC

**Figure 3:**
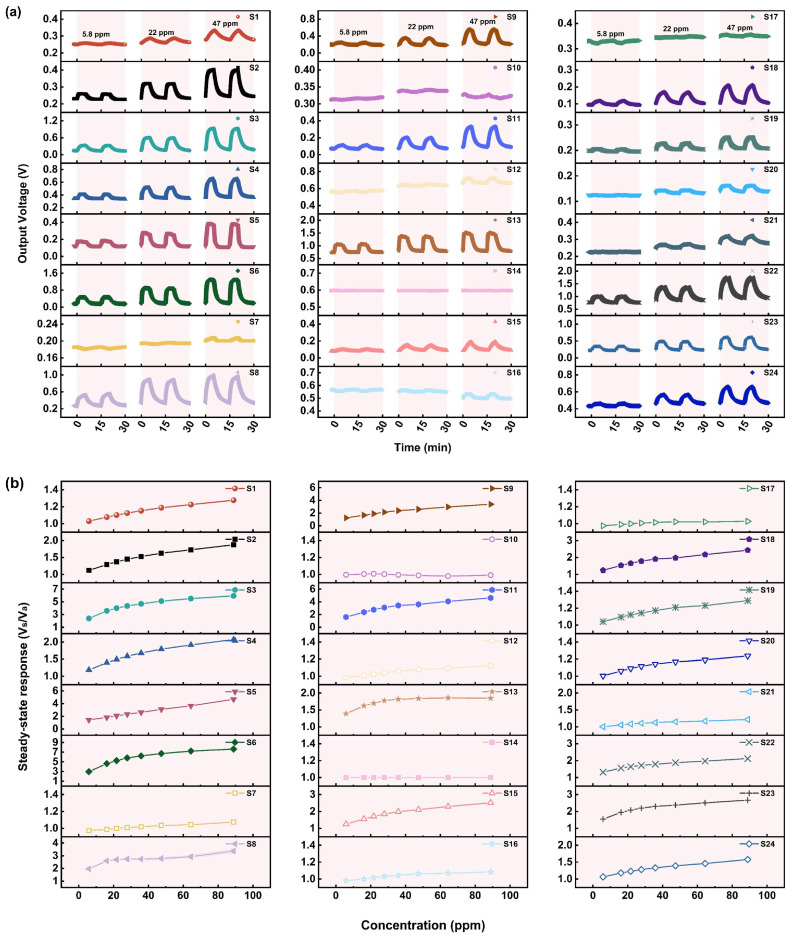
(**a**) The response curves of 24 MOS-based MEMS sensors to different concentrations of AC. (**b**) The relationship between the steady-state response and AC concentration (concentration of AC: 5.8–89 ppm; time for response: 5 min).

Based on the immediately dangerous for life and health (IDLH) level of AC (50 ppm), the gas sensing performance of the 24 MOS-based MEMS sensors for AC in a concentration range of 5.8–89 ppm was investigated. Figure 3a presents the response–recovery curves of the sensors exposed to different concentrations of AC, while Figure 3b demonstrates the relationship between the steady-state response (SR) and AC concentration.

It can be seen that more than half of the sensors, including S2–S6, S8, S9, S11, S13, S18, S19, and S22–S24, show a significant response to AC. Since the actual minimum detection concentration (5.8 ppm) is much lower than the IDLH level of AC (50 ppm), it can be assumed that these sensors have a low detection limit and high sensitivity for AC. The reasons for the significant responses may be as follows. Firstly, there may be many active sites on the MOS’s surface due to its high specific surface area. In addition, the steric hindrance of AC is weak due to its small size, resulting in a high coverage and large adsorption capacity of AC on the surface. Figure 3b also shows that the SR of the sensors increases nearly linearly as the AC concentration increases, which suggests their ability to detect a wider range of concentrations of AC, thus realizing quantitative detection.

Among the sensors that respond significantly to AC, most of them have a faster response time of mostly 1–3 min, but a longer recovery time. In terms of the recovery rate, except for S5, which has a recovery time of about 1 min, the recovery time of other sensors is about 4 min, and sometimes reaches 5–8 min. However, Figure 3a shows that the sensors can basically return to the baseline level when exposed to air again, and no significant shift in the signal baseline is observed after multiple response–recovery cycles, which indicates a good recovery ability. Although the groups generated by the interaction of AC with the sensing material may be strongly bonded to the surface and thus be difficult to desorb or dissociate, resulting in a slow recovery rate, the active sites on the MOS’s surface can be gradually released under the effects of high heating temperature and sufficient oxygen in clean air, and O_2_ will be chemically adsorbed to regenerate oxygen species, leading to a gradual return of the MOS’s resistance.

Based on the experimental results, it is speculated that the general evolution process AC on the MOS surface may occur as shown in Equation (1). AC combines with adsorbed oxygen species and reacts to form NO_2_, CO_2_, and H_2_O, which causes a large number of electrons to be transferred from the adsorbed molecules to the conduction band of the MOS, meaning that the sensor responds significantly. Moreover, nearly no ${\mathrm{CN}}^{-}$ is produced during the process that could stably bind to the MOS surface and cause a “poisoning effect”, and, therefore, the sensor exhibits excellent recovery performance [5].

(1) $$2\mathrm{HCN}+9O_{\left(\mathrm{ads}\right)}^{-}\to 2{\mathrm{NO}}_{2}+2{\mathrm{CO}}_{2}+H_{2}O+9e^{-}$$

#### 3.1.2. Performance of the Sensors in Relation to GB

**Figure 4:**
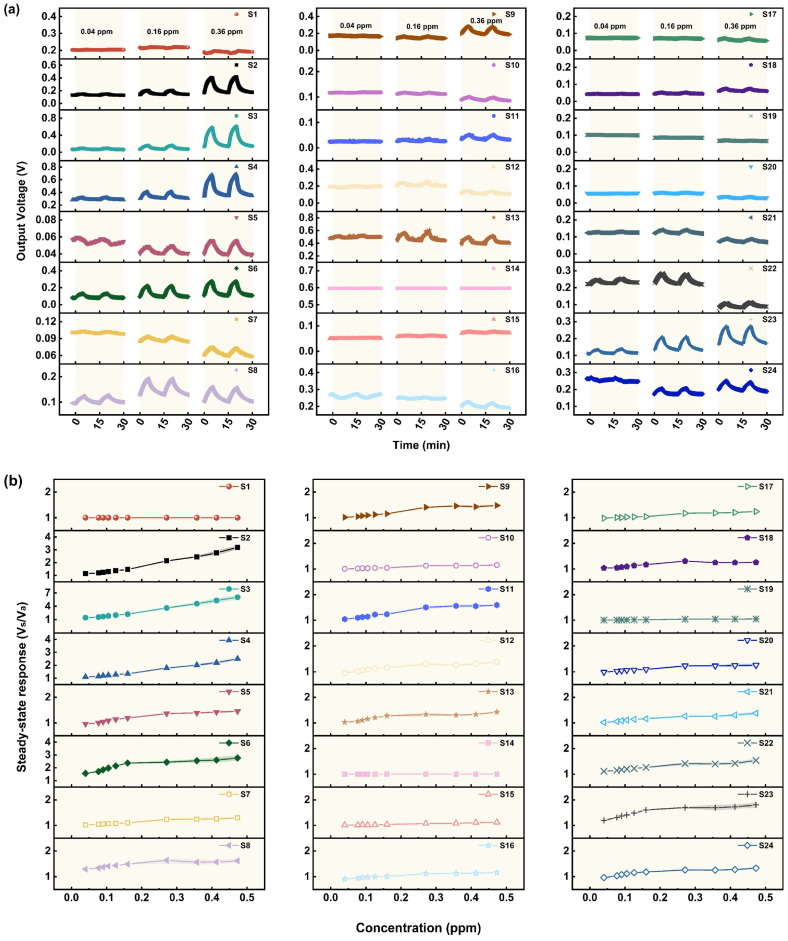
(**a**) The response curves of 24 MOS-based MEMS sensors to different concentrations of GB. (**b**) The relationship between the steady-state response and GB concentration (concentration of GB: 0.04–0.47 ppm; time for response: 5 min).

The gas sensing performance of 24 MOS sensors for different concentrations of GB (0.04–0.47 ppm) was studied, and the results are shown in Figure 4a,b. It is found that only a few sensors, including S2–S4, S6, S8, and S23, show a relatively significant response to GB. Since the actual minimum concentration (0.04 ppm) tested in the experiments is lower than the IDLH level of GB (0.05 ppm), it can be assumed that these sensors have a low detection limit and a high sensitivity to GB. Similarly to the results of the AC tests, it can be indicated from Figure 4b that sensors S2–S4 have the ability to detect a broader range of concentrations of GB and have the potential to realize quantitative detection. However, for other sensors with a weak response, the SR growth curve finally plateaued, indicating that the adsorption of GB on the MOS’s surface tended to be saturated. What should be pointed out is that the baseline of some sensors, such as S7, S8, S16, and S21–S24, fluctuates when testing different concentrations of GB. This may be due to differences in the properties of the carrier gas during the test, such as oxygen content and humidity. What is more, the properties of the MOS materials also affect the sensing performance.

The response rate of the sensors to GB is relatively slow compared to that of AC. Among the sensors that show a significant response to GB, S6 has a response time of about 3 min, while that of the other sensors is 3.5–4.5 min. This may be attributed to the steric hindrance effect because of the larger size of GB molecules. Therefore, the overall rate of adsorption, reaction, and electron transfer is slow, and the time required to reach the adsorption–reaction equilibrium is greater. In addition, the recovery rate of the sensors is also slow: the recovery times of S2–S4 and S6 are less than 5 min, but those of the other sensors are 5–9 min. This may be due to the slow desorption rate of P- and F-containing groups generated during the sensing process, which will probably result in a cumulative “poisoning effect” on the MOS’s surface [22,24]. When the sensors are re-exposed to clean air for a sufficient time, the signal of most sensors gradually returns to close to the baseline before exposure to GB.

The SR of sensors S2–S4 always shows a linear growth trend as the GB concentration increases from 0.04 ppm to 0.47 ppm. However, for sensors S6, S8, and S23 and other sensors with weak response to GB, their steady-state response grows linearly with concentration only when the concentration of GB is low, at 0.04–0.16 ppm. Then, the growth trend flattens with a further increase in GB concentration, which indicates saturated adsorption.

According to the aforementioned experimental results and the gas sensing mechanism of the GB simulant [6], i.e., DMMP, as well as the reaction mechanisms of GB hydrolysis and pyrolysis [34]. The possible evolution processes of GB on the MOS’s surface are shown in Equations (2)–(4). In this model, GB combines with adsorbed oxygen species first and reacts to produce isopropyl methylphosphonate and free fluoride ions adsorbed onto metal ions. It then further produces methyl phosphate, CO_2_, H_2_O, etc., and electrons are subsequently released to the conduction band of the MOS, resulting in a decrease in the resistance. The formed P-containing groups and $F^{-}$ may occupy the active sites and probably result in the slow recovery of the sensing material because they do not easily desorb or dissociate from the MOS’s surface.

(2) $${\mathrm{CH}}_{3}{\mathrm{PO}[\mathrm{OCH}(\mathrm{CH}}_{3}{)}_{2}{]F}_{\left(\mathrm{ads}\right)}+O_{\left(\mathrm{ads}\right)}^{-}\to {\mathrm{CH}}_{3}{\mathrm{PO}}_{2}{{[\mathrm{OCH}(\mathrm{CH}}_{3}{)}_{2}]}_{\left(\mathrm{ads}\right)}+F^{-}$$

(3) $${\mathrm{CH}}_{3}{\mathrm{PO}}_{2}{{[\mathrm{OCH}(\mathrm{CH}}_{3}{)}_{2}]}_{\left(\mathrm{ads}\right)}+O_{\left(\mathrm{ads}\right)}^{-}\to {\mathrm{CH}}_{3}{\mathrm{PO}}_{3\left(\mathrm{ads}\right)}+{\mathrm{OCH}(\mathrm{CH}}_{3}{)}_{2(\mathrm{ads})}^{-}$$

(4) $${2\mathrm{OCH}(\mathrm{CH}}_{3}{)}_{2(\mathrm{ads})}^{-}+17O_{\left(\mathrm{ads}\right)}^{-}\to 6{\mathrm{CO}}_{2}+7H_{2}O+19e^{-}$$

#### 3.1.3. Performance of the Sensors in Relation to GD

**Figure 5:**
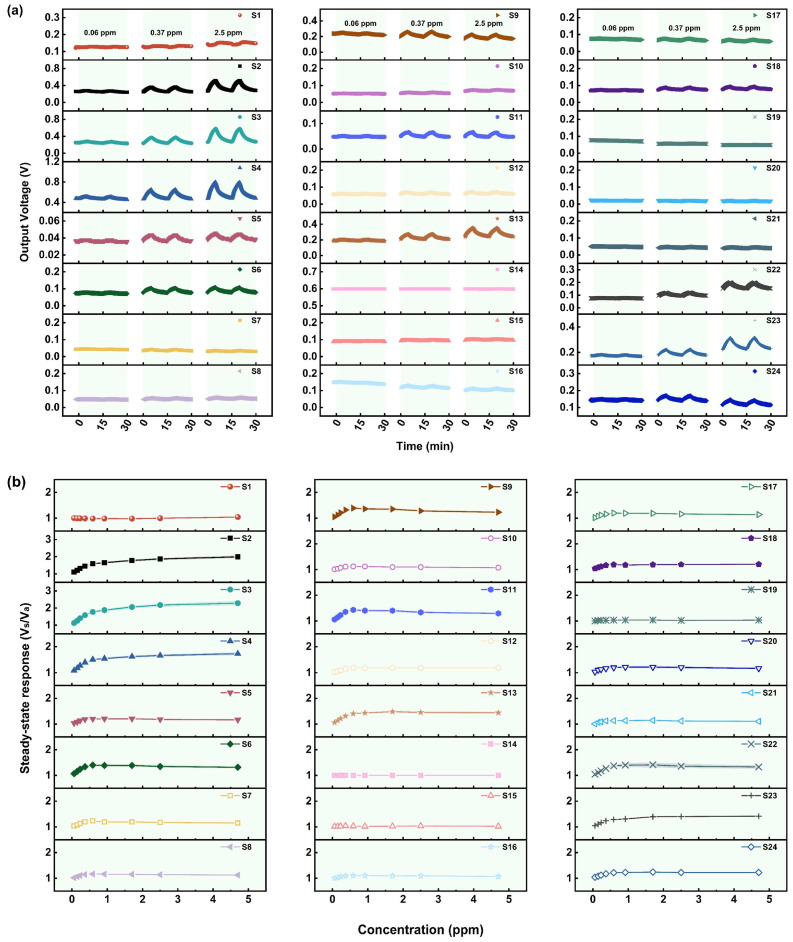
(**a**) The response curves of 24 MOS-based MEMS sensors to different concentrations of GD. (**b**) The relationship between the steady-state response and GD concentration (concentration of GD: 0.06–4.7 ppm; time for response: 5 min).

The gas sensing performance of 24 MOS sensors for different concentrations of GD (0.06–4.7 ppm) was studied. From the results shown in Figure 5a,b, it can be seen that fewer than half of the sensors, such as S2–S4, S6, S9, S11, S13, S22, and S23, display a relatively significant response. Considering that the IDLH level of GD is 0.008 ppm and lower than the actual detected minimum concentration, the theoretical LOD of the sensors was calculated. As Table S5 shows, the theoretical LOD of S2, S3, S4, and S6 is lower than 0.008 ppm, so these sensors have the ability to detect and provide warnings about GD under extremely low concentrations. In addition, it can be seen from Figure 5b that SR of the sensors first shows a linear growth trend with the increase in GD concentration, and then a plateau appears, indicating saturated adsorption.

The sensors have a response time of 3–5 min and a recovery time of 6–9 min. When the sensors are re-exposed to clean air for a sufficient time, they can recover to a high degree. This indicates that the active sites on the MOS’s surface are gradually released under the effects of high heating temperature and oxygen in the air. Therefore, the adsorbed oxygen species regenerate, and the resistance of the sensing materials is restored.

The SR of the sensors to GD is generally weaker, and the response rates are slower than those to GB. It may be because GD has a larger steric hindrance. As a result, it will take longer for GD to reach adsorption–reaction equilibrium, and the adsorption capacity will be lower.

Following the aforementioned experimental results and the gas sensing mechanism of the GD simulant [6] (i.e., DMMP, as well as reaction mechanisms of GD hydrolysis and pyrolysis [34]), the possible evolution processes of GD on the MOS surface are shown in Equations (5)–(7). In this model, GD firstly combines with adsorbed oxygen species and reacts to produce pinacate methylphosphonate and fluoride ions adsorbed onto metal ions, and then further produces methyl phosphate, CO_2_, H_2_O, etc. Consequently, electrons are released to the conduction band, resulting in a decrease in the resistance. Similarly to GB, the P-containing groups and $F^{-}$ generated during the GD sensing process may occupy the active sites and do not easily desorb or dissociate from the MOS surface, probably resulting in a slow recovery rate.

(5) $${\mathrm{CH}}_{3}{\mathrm{PO}[\mathrm{OCH}(\mathrm{CH}}_{3})({C(\mathrm{CH}}_{3}{)}_{3}){]F}_{\left(\mathrm{ads}\right)}+O_{\left(\mathrm{ads}\right)}^{-}\to {\mathrm{CH}}_{3}{\mathrm{PO}}_{2}{{[\mathrm{OCH}(\mathrm{CH}}_{3})({C(\mathrm{CH}}_{3}{)}_{3})]}_{\left(\mathrm{ads}\right)}+F^{-}$$

(6) $${\mathrm{CH}}_{3}{\mathrm{PO}}_{2}{{[\mathrm{OCH}(\mathrm{CH}}_{3}){C(\mathrm{CH}}_{3}{)}_{3}]}_{\left(\mathrm{ads}\right)}+O_{\left(\mathrm{ads}\right)}^{-}\to {\mathrm{CH}}_{3}{\mathrm{PO}}_{3\left(\mathrm{ads}\right)}+{\mathrm{OCH}(\mathrm{CH}}_{3}){C(\mathrm{CH}}_{3}{)}_{3(\mathrm{ads})}^{-}$$

(7) $${2\mathrm{OCH}(\mathrm{CH}}_{3}){C(\mathrm{CH}}_{3}{)}_{3(\mathrm{ads})}^{-}+35O_{\left(\mathrm{ads}\right)}^{-}\to 12{\mathrm{CO}}_{2}+13H_{2}O+36e^{-}$$

#### 3.1.4. Performance of the Sensors in Relation to VX

**Figure 6:**
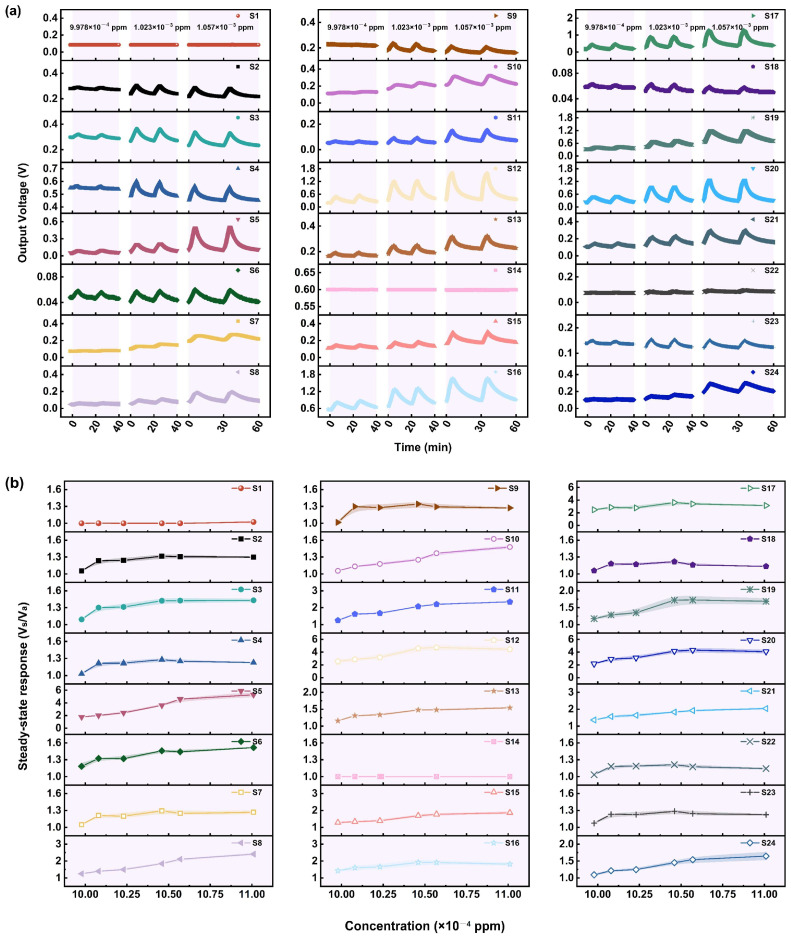
(**a**) The response curves of 24 MOS-based MEMS sensors to different concentrations of VX. (**b**) The relationship between the steady-state response and VX concentration (concentration of VX: 9.978 × 10^−4^–1.101 × 10^−3^ ppm; time for response: 5 min).

The VX concentrations achieved by the experimental system range from 9.978 × 10^−4^ to 1.101 × 10^−3^ ppm, which are lower than the IDLH level of VX, 0.004 ppm. The gas sensing performance of the 24 MOS sensors for different concentrations of VX was studied and the results are shown in Figure 6a,b. Fortunately, despite the low VX concentration, most of the sensors in the array, including S2–S6, S8, S11–S13, S15–S17, S19–S21, S23, and S24, show a significant response to VX, enabling qualitative detection. In addition, most of these sensors show a response of more than 1.5, even 2.5, at the lowest measured concentration of VX, suggesting their ability to qualitatively detect VX at low concentrations.

The SR of the sensors with a significant response to VX firstly shows a linear growth trend as the VX concentration increases form 9.978 × 10^−4^ to 1.046 × 10^−3^ ppm. When VX concentration increases over 1.05 × 10^−3^ ppm, the growth trend of the steady-state response becomes flat, suggesting a saturation of the adsorption of VX. However, it is difficult to apply the sensor array in the quantitative detection of VX, because the VX concentration measured in this work is extremely low, and the sensor response will be greatly affected by the baseline, environment, and other factors. Regarding the abovementioned issues, more in-depth research needs to be carried out. For example, the dynamic gas distribution system should be improved to obtain VX with a higher concentration, and the gas sensing materials should be modified or the device structures should be optimized to enhance the baseline stability and anti-interference ability of the sensors. These are possibly important directions for our future work.

The sensors also show a slow response and recovery rate. The response times of the sensors are mostly over 4 min, and the recovery time is over 8 min. After multiple response–recovery cycles, the sensor baseline shifts to a certain extent, and it takes a long time to recover to near the pre-response baseline. The reasons for the slow response rate to VX may be attributed to it having the largest steric hindrance among the CWAs containing P atoms. In addition, there may be multiple bonds that can be broken in VX molecules, meaning that the gas sensing process of VX on the MOS’s surface may be completed in multiple steps. Meanwhile, the poor recovery ability of the sensors may be related to the strong bonding of P-, S-, and N-containing groups generated during the gas sensing process.

According to the experimental results and the reaction mechanisms of VX hydrolysis and pyrolysis [34], the possible evolution processes of VX on MOS surface are shown in Equations (8)–(11). First, VX is decomposed into two free radicals at high temperatures according to Equation (8), namely ${\mathrm{CH}}_{3}{\mathrm{PO}(\mathrm{OC}}_{2}H_{5})\cdot$ and $\cdot {\mathrm{SCH}}_{2}{\mathrm{CH}}_{2}{N[\mathrm{CH}(\mathrm{CH}}_{3}{)}_{2}{]}_{2}$. These are further combined with the adsorbed oxygen species to initiate surface reactions, as shown in Equations (9)–(11), generating CO_2_, SO_2_, NO_2_, H_2_O, etc., and releasing large amounts of electrons to the conduction band of MOS materials. This may be one of the reasons why the sensors respond significantly to VX. Meanwhile, the P-, S-, and N-containing groups generated during the processes may occupy the active sites including metal ions, being difficult to desorb or dissociate from the MOS’s surface, resulting in a slow recovery rate.

(8) $${\mathrm{CH}}_{3}{\mathrm{PO}(\mathrm{OC}}_{2}H_{5}{)\mathrm{SCH}}_{2}{\mathrm{CH}}_{2}{N[\mathrm{CH}(\mathrm{CH}}_{3}{)}_{2}{]}_{2(\mathrm{ads})}\to {\mathrm{CH}}_{3}{\mathrm{PO}(\mathrm{OC}}_{2}H_{5})\cdot +\cdot {\mathrm{SCH}}_{2}{\mathrm{CH}}_{2}{N[\mathrm{CH}(\mathrm{CH}}_{3}{)}_{2}{]}_{2}$$

(9) $${\mathrm{CH}}_{3}{\mathrm{PO}(\mathrm{OC}}_{2}H_{5})\cdot +O_{\left(\mathrm{ads}\right)}^{-}\to {\mathrm{CH}}_{3}{\mathrm{PO}}_{3\left(\mathrm{ads}\right)}+{\mathrm{OC}}_{2}H_{5(\mathrm{ads})}^{-}$$

(10) $${2\mathrm{OC}}_{2}H_{5(\mathrm{ads})}^{-}+11O_{\left(\mathrm{ads}\right)}^{-}\to 4{\mathrm{CO}}_{2}+5H_{2}O+13e^{-}$$

(11) $$\cdot {\mathrm{SCH}}_{2}{\mathrm{CH}}_{2}{N[\mathrm{CH}(\mathrm{CH}}_{3}{)}_{2}{]}_{2}+29O_{\left(\mathrm{ads}\right)}^{-}\to {\mathrm{SO}}_{2}+8{\mathrm{CO}}_{2}+{\mathrm{NO}}_{2}+9H_{2}O+29e^{-}$$

#### 3.1.5. Performance of the Sensors in Relation to HD

**Figure 7:**
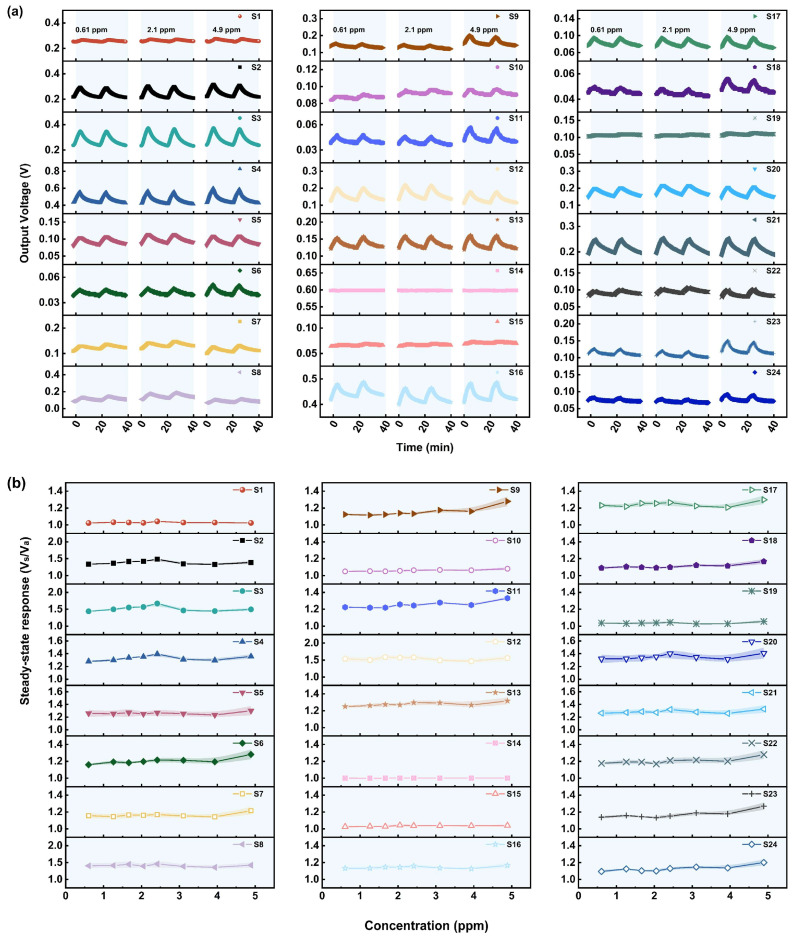
(**a**) The response curves of 24 MOS-based MEMS sensors to different concentrations of HD. (**b**) The relationship between the steady-state response and HD concentration (concentration of HD: 0.61–4.9 ppm; time for response: 5 min).

The gas sensing performance of 24 MOS sensors for different concentrations of HD (0.61–4.9 ppm) was studied. From the results shown in Figure 7a,b, it can be seen that most sensors, including S2–S6, S8, S9, S11–S13, S16–S18, and S20–S24, show a significant response to HD. Considering that the IDLH level of HD is 0.1 ppm and is much lower than the actual minimum detection concentration, the theoretical LOD of the sensors was calculated, and the results are shown in Table S6. The theoretical LOD of S2–S4 is less than 0.1 ppm, thus meeting the needs for detecting HD under low concentrations.

Figure 7b shows that the HD adsorption capacity on the MOS’s surface may have become saturated in the range of HD concentrations measured in this work. The response and recovery rate of the sensors is slow, as the response time of the sensors is mostly longer than 4 min, and the recovery time is 9–10 min. The reason for the slow response may be that the energy barrier that needs to be overcome is high for HD molecules reacting with adsorbed oxygen species. Meanwhile, the slow recovery rate may be attributed to the strong bonding between Cl-containing groups and the MOS’s surface. When the sensors are re-exposed to clean air for a sufficient time, they can recover to a high degree. This suggests that the active sites on the MOS’s surface can be slowly released, and thereby the oxygen species are regenerated.

According to the aforementioned experimental results and the gas sensing mechanism of the HD simulant [35,36], i.e., 2-CEES, as well as the degradation mechanism of HD, the possible evolution processes of HD on the MOS’s surface are shown in Equations (12)–(14). First, HD is decomposed into two free radicals at a high temperature according to Equation (12), namely ${\mathrm{ClCH}}_{2}{\mathrm{CH}}_{2}\cdot$ and ${\mathrm{ClCH}}_{2}{\mathrm{CH}}_{2}S\cdot$. They have the properties of a Lewis base and therefore can adsorb to the Lewis acidic metal ions on the MOS’s surface and react with the adsorbed oxygen species, as shown in Equations (13) and (14). Finally, this will generate CO_2_, Cl_2_, SO_2_, H_2_O, etc.

(12) $${\mathrm{ClCH}}_{2}{\mathrm{CH}}_{2}{\mathrm{SCH}}_{2}{\mathrm{CH}}_{2}{\mathrm{Cl}}_{\;(\mathrm{ads})}\to {\mathrm{ClCH}}_{2}{\mathrm{CH}}_{2}\cdot +{\mathrm{ClCH}}_{2}{\mathrm{CH}}_{2}S\cdot$$

(13) $${2\mathrm{ClCH}}_{2}{\mathrm{CH}}_{2}\cdot +12O_{\left(\mathrm{ads}\right)}^{-}\to 4{\mathrm{CO}}_{2}+{\mathrm{Cl}}_{2}+4H_{2}O+12e^{-}$$

(14) $${2\mathrm{ClCH}}_{2}{\mathrm{CH}}_{2}S\cdot +16O_{\left(\mathrm{ads}\right)}^{-}\to 2{\mathrm{SO}}_{2}+{\mathrm{Cl}}_{2}+4{\mathrm{CO}}_{2}+4H_{2}O+16e^{-}$$

#### 3.1.6. Potential Applications and Limitations of the Sensors

The qualitative comparisons of the response performance of 24 sensors to five CWAs are listed in Table S7. According to the aforementioned results and Table S7, the potential applications and limitations of the proposed sensors can be summarized as follows.

**Figure 8:**
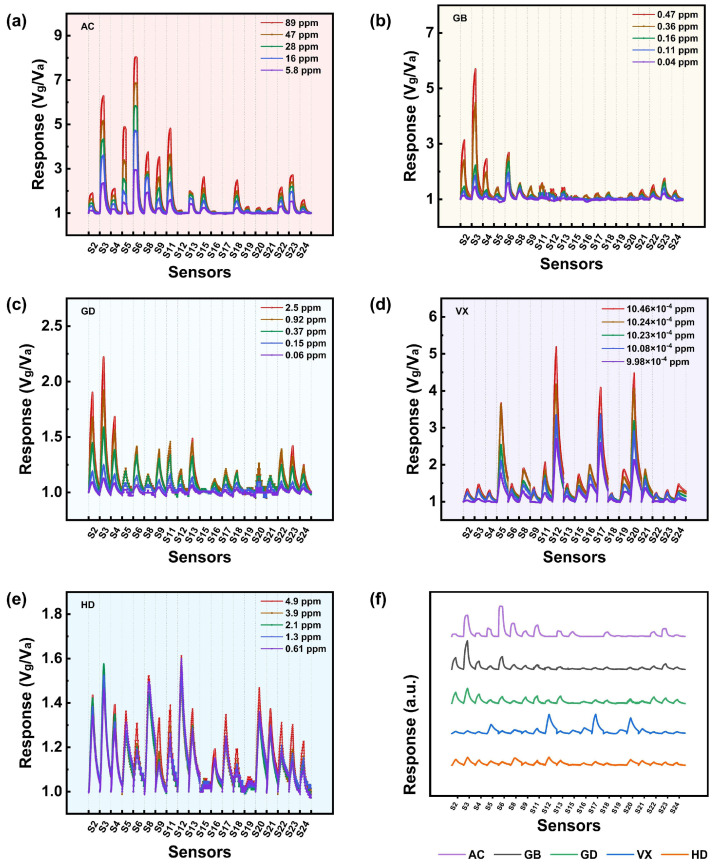
The visualization pattern of the response–recovery signal of 20 sensors towards five CWAs. Patterns of (**a**) AC, (**b**) GB, (**c**) GD, (**d**) VX, and (**e**) HD at 5 concentrations. (**f**) Graphical comparison of the response signals and recovery of 20 sensors in relation to five CWAs.

S1, S7, S10, and S14 exhibit weak or nearly no response to the five CWAs, but the remaining 20 sensors exhibit good gas sensing performance, such as a relatively significant response, a low detection limit, and a broad detection range, towards CWAs in different combinations. For example, S2–S4, S6, S8, and S23 show good performance in GB sensing, while S2–S4, S6, S9, S11, S13, S22, and S23 show good performances in GD sensing. Although a single sensor did not exhibit selectivity towards a specific CWA, the abovementioned differences in the combination provide the possibility for discriminating between the five CWAs. This is probably attributed to both the different composition, morphology, and structure of the sensing materials of the 20 sensors, which are mainly different forms of modified SnO_2_, and the different molecular structure and properties of each CWA. Therefore, the adsorption and reaction of each CWA on different MOS surfaces are also distinguished. Accordingly, the response–recovery signals of the 20 sensors, excluding S1, S7, S10, and S14, to the five CWAs are visualized in Figure 8, and it can be seen that each CWA corresponds to a specific pattern. Based on this, features could be extracted from the testing data to construct feature datasets for further pattern recognition of the five CWAs, which will be discussed later. The sensors still work normally after undergoing gas sensing tests spanning over one year, which indicates good stability and a long working life in practical applications.

However, it should be noted that the recovery rate of some sensors after responding to CWAs is relatively slow, although the sensors can gradually return to the baseline after exposure to clean air for a long time. In addition, the moisture resistance and anti-interference ability of the sensors have not been investigated. Moreover, the actual sensing mechanisms of each CWA on MOS surfaces, rather than the speculated reactions, have not been studied in this work. These problems will limit the application of these sensors. Therefore, conducting further in-depth research to reveal or solve these problems will be one of the important directions in our future work.

### 3.2. Pattern Recognition

#### 3.2.1. Kinetic Characteristics and Principal Component Analysis

From the dynamic response signals of 20 sensors to five CWAs, it is found that the sensor response grows slowly when the target gas is introduced for 10–30 s and then there is a rapid and significant increase after the target gas has been introduced for 30–50 s, and that the signal change in the initial stage of the response is firstly slow and then fast and most of the response curves are S-shaped. This is probably due to both the dispersion in the gas mixing chamber and molecular diffusion transport onto the MOS’s surface. In the dynamic gas distribution system, it takes time for the CWA gas flow in the test chamber to reach a stable concentration and an entry–exit equilibrium, meaning that the signal is weak in the early stage of the sensor response. The response rate of the sensors to the five CWAs may be closely related to their molecular diffusion behavior in air alone and on the MOS’s porous surface, as well as the collision, binding, and reaction behaviors of the molecules with the surface active sites, meaning that the response rate of each sensor to each CWA is different. Based on this, features are extracted from the kinetic characteristics of the sensor’s response stage and are used as the basis for the classification in principal component analysis (PCA).

As shown in Figure S1, the output signals of the sensors when exposed to CWAs for 10–30 s and 30–50 s were extracted, and the corresponding slopes were obtained by means linear fitting, denoted as k_C1_ and k_C2_, respectively, to represent the diffusion–reaction rate of CWAs in the primary stage of the response on MOS surfaces to a certain extent. The k_C1_ and k_C2_ of the response curves of 20 sensors to five CWAs at different concentrations were extracted sequentially, and finally, a dataset containing 40 features and 273 samples was constructed, denoted as P.

**Figure 9:**
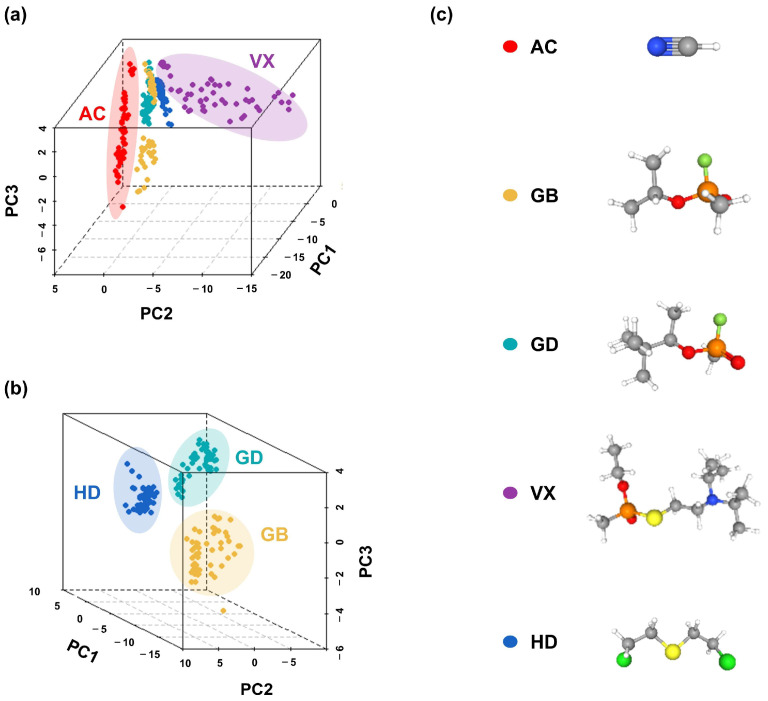
PCA classification scatter plots based on features extracted from the kinetic characteristics (k_C1_, k_C2_) of 20 sensors’ response to the five CWAs. (**a**) PCA classification effects of the five CWAs. (**b**) PCA classification effects of GB, GD, and HD. (**c**) The chemical structures of the five CWAs and their corresponding symbol colors.

PCA was carried out based on dataset P, and the resulting classification effects are shown in Figure 9. As can be seen from Figure 9a, the data points attributed to AC and VX form a relatively independent group; meanwhile, there is some degree of overlap between the data points attributed to GB, GD, and HD. PCA was further carried out based on the features derived from GB, GD, and HD, obtaining Figure 9b, and the data points for GB, GD, and HD were found to, in fact, be aggregated to form a separate group. In summary, the five tested CWAs can be preliminarily distinguished based on the features extracted from the kinetic characteristics of the sensor′s response and through the PCA method.

#### 3.2.2. Dynamic Change Characteristics and Machine Learning Algorithms

Although the kinetic characteristics of k_C1_ and k_C2_ in the response stage can reflect the adsorption–reaction rate between CWAs and the MOS material to a certain extent, the computational effort required to extract them is great. In response to this problem, the signal data in the response stage of 20 sensors for each CWA were extracted to form visual graphics, and their features based on the specific patterns were utilized as the basis to identify the five CWAs. The extraction of such features only relies on the original data, which has the advantages of simple data processing and easy implementation using an algorithm.

However, considering that the frequency of data collection by the sensors is 1 Hz, if all the data points in the 20 sensor response stages were taken as 1 sample, the number of data points in the sample would be about 6000. Such a large sample would not only cause redundancy but also increase the computational burden. Therefore, a periodic interval sampling method was adopted at the same time to extract five subsets from one sample. As shown in Figure S2, each subset contained about 10 data points, which were extracted from the response stage of each sensor, and the patterned curves formed by the data points of 20 sensors reflected the changes in the original response signal of sensors to CWAs. The 267 × 5 subsets were combined into a new feature dataset, denoted as N. The dataset N contained 200 features and 1365 samples.

**Figure 10:**
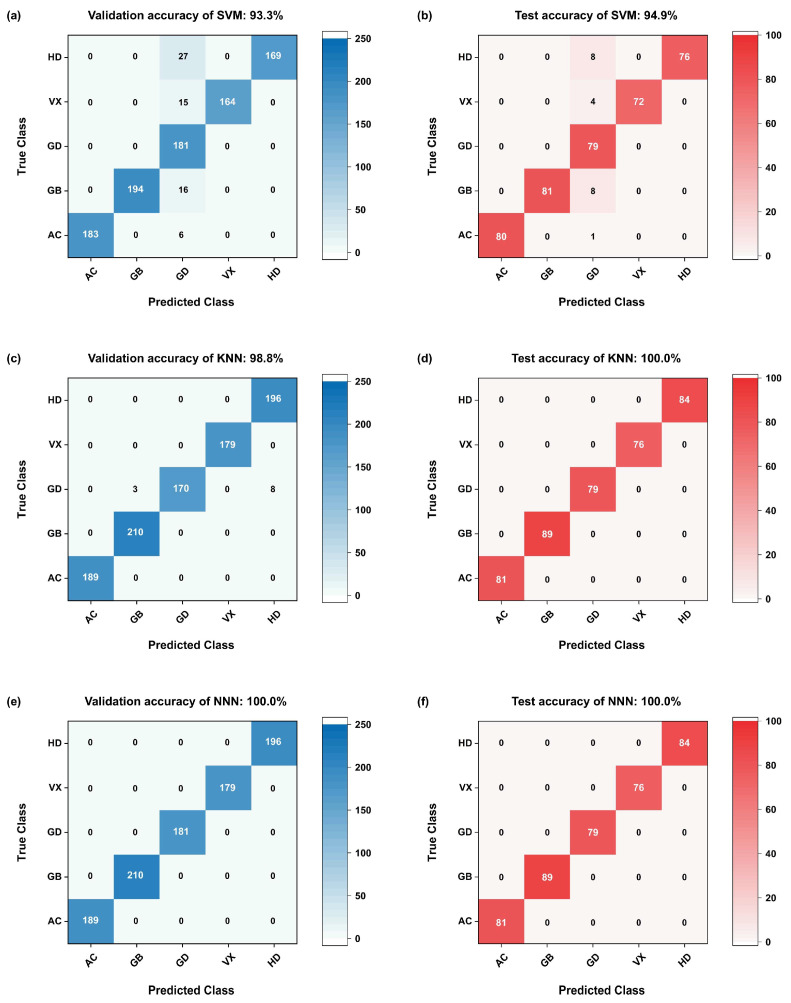
Classification effect of five CWAs through different machine learning algorithms based on features extracted from the dynamic change characteristics of the signal in the response stage. (**a**,**b**) SVM; (**c**,**d**) KNN; (**e**,**f**) NNN.

Although the PCA method can be used to identify CWAs based on the dataset P, the underlying logic of the data needs to be linear, and PCA is not suitable for processing datasets with larger amounts of data and more complex features. Therefore, for the dataset N, typical machine learning algorithms with better generalization ability, simpler learning rules, and greater computer compatibility were applied to achieve gas identification, such as support vector machine (SVM), the K-nearest neighbor algorithm (KNN), and the narrow neural network (NNN), where the NNN has only one hidden layer and the model is relatively simple. The results are shown in Figure 10. It can be seen that the validation accuracy of SVM, KNN, and NNN is 93.3%, 98.8%, and 100%, respectively, and the test accuracy is 94.9%, 100.0%, and 100.0%, respectively. Apparently, NNN displays the highest accuracy and the best classification effect. In summary, the five CWAs can be accurately classified through an appropriate neural network model based on features extracted from the dynamic change characteristics of the signal in the response stage.

A comparison of the device structure, target gases, and classification effects of the MOS sensor array for CWA detection presented in this work and those in the literature [22,27,28] is presented in Table S8. The number of relevant studies is limited due to the high toxicity of the target gases, namely CWAs and their simulant agents, meaning that they are difficult to obtain and their use is restricted. Firstly, it can be seen that the sensors in the array studied in this work are smaller in size and exhibit lower power consumption because they are MEMS sensors fabricated based on silicon-based MHPs. In addition, the algorithms applied in this work, such as SVM, KNN, and NNN, can provide more specific digital recognition accuracy, rather than through patterning and visual discrimination based on the results of linear discriminant analysis (LDA) and PCA. Most importantly, the target gases studied herein are all actual CWAs, rather than simulants, indicating the great practical application potential of the presented MOS sensor array in the detection and discrimination of actual CWAs.

## 4. Conclusions

In this work, a low-power and small-sized sensor array composed of 24 MOS-based MEMS gas sensors was constructed. Firstly, the gas sensing performance of each sensor in the array in relation to five CWAs, namely AC, GB, GD, VX, and HD, was studied. For each CWA, there are some sensors in the array that show good gas sensing performance, including a relatively significant response, a low detection limit, and a broad detection range. However, it should be noted that due to limitations in the gas distribution system, we were unable to conduct a comparative analysis of the sensors’ performance in a real environment under varying temperature and humidity conditions. Therefore, optimizing the gas distribution system to carry out such research will be one of the key directions in our future work.

The sensor array exhibits unique signal patterns for different CWAs, probably due to the different sensing behaviors of each CWA on different MOS surfaces. The sensing mechanism is not clear and needs to be further investigated in the future. However, such unique signal patterns lay a good foundation for CWA classification. Based on this, features were extracted from the initial kinetic characteristics of the sensor response curve and the dynamic change characteristics of signals in the response stage. Accordingly, two corresponding feature datasets were constructed, and pattern recognition analysis was carried out using PCA and machine learning algorithms to realize the classification of the five CWAs. The 3D classification plots obtained by means of PCA showed significant differences between groups belonging to the five CWAs. Among the three machine learning algorithms, namely SVM, KNN, and NNN, NNN showed the best classification effects, with a validation accuracy and test accuracy both as high as 100.0%.

In conclusion, using MOS-based MEMS sensor arrays driven by pattern recognition algorithms can realize the detection and identification of CWAs. If such a sensor array is integrated into a portable device, it will show great potential and practical application value for the rapid and on-site detection of CWAs. This work is also of great practical significance in making up for the shortcomings of the existing techniques for CWA detection and is of benefit for the establishment of a more comprehensive technical system. In order to further enhance the application potential of this sensor array, it is necessary to investigate its anti-interference ability and its ability to simultaneously detect and classify mixtures of multiple CWAs and to train the pattern recognition algorithms based on more complex datasets to ensure their accuracy in the future.

## Data Availability

The original contributions presented in this study are included in the article/Supplementary Materials. Further inquiries can be directed to the corresponding authors.

## Supplementary Materials

The following supporting information can be downloaded at: https://www.mdpi.com/article/10.3390/s25082633/s1, S.I.1 Tables. Table S1: Advantages and disadvantages of conventional CWAs detection techniques; Table S2: Full name, molecular formula and chemical structures of five chemical warfare agents; Table S3: Sensing materials and heating voltage of the 24 MOS-based MEMS sensors; Table S4: The concentration range of CWAs during the gas sensing tests; Table S5: Theoretical LOD of the sensors for GD; Table S6: Theoretical LOD of the sensors for HD; Table S7: Qualitative comparison of the response performance of 24 sensors to 5 CWAs; Table S8: Comparison of the device structure, target gases and classification effect of MOS sensors array for detection of CWAs and their simulants in the literature and this work. S.I.2 Figures. Figure S1: The dynamic response-recovery curve of S3 respond to (a) AC at 5.8 ppm, (b) GB at 0.16 ppm, and schematic diagram of extracting features from the kinetic characteristics of response curves (k_C1_, k_C2_); Figure S2: The pattern of a single response-recovery cycle of 20 sensos to at AC 16 ppm and the patterns of 5 subsets obtained by periodic interval sampling. References [1,2,3,4,5,6,7,12,13,15,16,17,18,22,23,24,25,27,28] are cited in the supplementary materials.

## Abbreviations

The following abbreviations are used in this manuscript:

CWAs	Chemical warfare agents
MOS	Metal oxide semiconductor
MEMS	Micro-Electro-Mechanical System
AC	Hydrogen cyanide
GB	2-[fluoro(methyl)phosphoryl]oxypropane
GD	3-[fluoro(methyl)phosphoryl]oxy-2,2-dimethylbutane
VX	Ethyl S-(2-diisopropylaminoethyl) methylphosphonothioate
HD	Di-2-chloroethyl sulfide
PCA	Principal component analysis
LOD	Low limit of detection
R	Response
SR	Steady-state response
MR	Maximum response
IDLH	Immediately dangerous for life and health
SVM	Support vector machines
KNN	K-nearest neighbor algorithm
NNN	Narrow neural networks
LDA	Linear discriminant analysis
