# Supplementary material for: Detection and Pattern Recognition of Chemical Warfare Agents by MOS-Based MEMS Gas Sensor Array
Source: Sensors (Basel). 2025 Apr 21;25(8):2633. doi: 10.3390/s25082633 (PMC12030887; doi:10.3390/s25082633)
Supplement: Supplementary file 1 [file sensors-25-02633-s001.zip › sensors-3546144-supplementary.pdf]

Supporting Information

Detection and Pattern Recognition of Chemical Warfare Agents by MOS-Based MEMS Gas Sensor array

S.I.1 Tables

Table S1 Advantages and disadvantages of conventional CWAs detection techniques.

Detection techniques	Advantages	Disadvantages
Gas chromatography, Gas chromatography-Mass spectrometer[1,2]	Minimal sample preparation; accurate analysis; sensitive and reliable.	Bulk and expensive; time consuming; high vapour pressure needed; unable to do direct analysis of arsenic compounds; qualified personnel required.
Liquid chromatography[1,2]	High separation capacity of complex samples; strong qualitative ability.	Isobaric interference; ion suppression effect; unpredictable ion yield attenuations; expensive.
Ion mobility spectrometry[1,2]	Lightweight; low power consumption; sensitive; short analysis time required; portable.	Limited selectivity; high false alarm rates; radioactive source required; affected by fluctuations in temperature, pressure and humidity.
Infrared spectroscopy[1,2]	High sensitivity; fast detection and response; low limit of detection; non- destructive sample analysis.	High cost; complexity; large size; sensitive to vibration.
Electrochemical sensing technology[3,4]	Rapid response; easy operation; high sensitivity; high-precision; high selectivity; low cost.	Limited temperature range; limited shelf life.
Surface acoustic wave sensors[4]	Diverse range of coatings; high sensitivity; fast response; ease integration.	Difficult to reproduce complex interface circuitry.
Silicon nanowire sensors [5,6]	High sensitivity.	Incomplete reversibility.
Quartz crystal microbalance (QCM) sensors[7,8]	High sensitivity.	Low recovery rate.
Metal oxide semiconductor (MOS) sensors[9–17]	Low cost, fast response time; wide range of target gases, ease miniaturization; easy operation; portable.	Relatively low sensitivity and selectivity.

Table S2 Full name, molecular formula and chemical structures of five chemical warfare agents

CWAs (Codes)	Full name	Molecular formula	Chemical structures
AC	Hydrogen cyanide	HCN	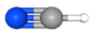
Sarin (GB)	2-[fluoro(methyl)phosphoryl]oxypropane	$C_4H_{10}FO_2P$	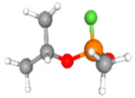
Soman (GD)	3-[fluoro(methyl)phosphoryl]oxy-2,2-dimethylbutane	$C_7H_{16}FO_2P$	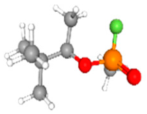
VX	Ethyl S-(2-diisopropylaminoethyl) methylphosphonothioate	$C_{11}H_{26}NO_2PS$	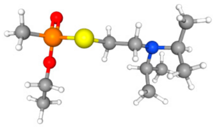
Mustard gas (HD)	Di-2-chloroethyl sulfide	$C_4H_8Cl_2S$	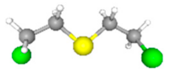

Table S3 Sensing materials and heating voltage of the 24 MOS-based MEMS sensors

Sensor	Sensing material	Heating voltage (V)
S1	SnO_2-5%Pd	5
S2	WO_3-Pt	5
S3	SnO_2-100 nm	5
S4	SnO_2-Fe	5
S5	SnOx-10 nm (x<2)	1.8
S6	WO_3	1.8
S7	SnO_2+1%Pt+1%Pd	1.8
S8	SnO_2-50 nm	2.2
S9	SnO_2-5%Ti	2.2
S10	50%WO_3-50%SnO_2-2%Pd	2.5
S11	SnO_2-2%Al-2%Si	1.8
S12	20%ZnO-80%SnO_2	1.8
S13	SnO_2-3%Fe	1.8
S14	SnO_2-20 nm	2.2
S15	ZnO-4%Pt	2.2
S16	90%SnO_2-10%Al_2O_3-2%Pt	2.5
S17	SnO_2+5%Pt+5%Pd	1.8
S18	SnO_2+2%Fe+3%Co	1.8
S19	ZnO-2%Pt-2%Pd	2.0
S20	SnO_2-5%Pt-2%Pd	2.0
S21	SnO_2-2%Pt-1%Si	2.5
S22	SnO_2-Pt	2.5
S23	SnO_2-2%Pt	3.3
S24	SnO_2-2%Pd	2.8

Table S4 The concentration range of CWAs during the gas sensing tests

CWAs	IDLH (ppm)	Concentration range (ppm)
AC	50	5.8 ~ 89
GB	0.05	0.04 ~ 0.47
GD	0.008	0.06 ~ 4.7
VX	0.004	9.978×10^{-4} ~ 1.101×10^{-3}
HD	0.1	0.61 ~ 4.9

Table S5 Theoretical LOD of the sensors for GD

Sensors	LOD (ppm)	Sensors	LOD (ppm)
S2	0.0024	S11	0.032
S3	0.0018	S13	0.015
S4	0.0021	S22	0.027
S6	0.0045	S23	0.013
S9	0.0060		

IDLH of GD is 0.008 ppm. Only the theoretical LOD for the sensors with a significant response to GD are listed in the table.

Table S6 Theoretical LOD of the sensors for HD

Sensors	LOD (ppm)	Sensors	LOD (ppm)
S2	0.061	S12	0.40
S3	0.042	S13	0.41
S4	0.060	S16	0.22
S6	0.34	S20	0.47
S8	0.52		

IDLH of HD is 0.1 ppm. Only the theoretical LOD for the sensors with a significant response to HD are listed in the table.

Table S7 Qualitative comparison of the response performance of 24 sensors to 5 CWAs

Sensors	Response performance to CWAs				
	AC	GB	GD	VX	HD
S1	○	○	○	○	○
S2	√	√	√	√	√
S3	√	√	√	√	√
S4	√	√	√	√	√
S5	√	○	○	√	√
S6	√	√	√	√	√
S7	○	○	○	○	○
S8	○	√	○	√	√
S9	√	○	√	○	√
S10	○	○	○	○	○
S11	√	○	√	√	√
S12	○	○	○	√	√
S13	√	○	√	√	√
S14	○	○	○	○	○
S15	○	○	○	√	○
S16	○	○	○	√	√
S17	○	○	○	√	√
S18	√	○	○	○	√
S19	√	○	○	√	○
S20	○	○	○	√	√
S21	○	○	○	√	√
S22	√	○	√	○	√
S23	√	√	√	√	√
S24	√	○	○	√	√

The symbol "√" represents a relatively significant response, while the symbol "○" represents a relatively weak response or no response.

Table S8 Comparison of the device structure, target gases and classification effect of MOS sensors array for detection of CWAs and their simulants in literature and this work

Reference	Device			Target gases		Pattern recognition	
	Substrate	Size of one sensor	Power consumption of one sensors in average	Category	Detection range	Method	Effect
[16]	Alumina	3 mm × 3 mm	NA	DCP; DMMP	DCP: 3 ppm; 10 ppm<br>DMMP: 0.5 ppm; 5 ppm	LDA	Good discrimination
[18]	Alumina	2 mm × 2 mm	500 mW	DMMP; ACN; DCM	0 ~ 3 ppm	PCA	Easy classification
[19]	Silicon	NA	NA	DCP; DCNP; DFP	0.08 ~ 208 ppm	PCA	Effective discrimination
This work	Silicon	0.9 mm×0.9 mm	<10 mW	AC; GB; GD; VX; HD	AC: 5.8~89 ppm;<br>GB: 0.04~0.47 ppm;<br>GD: 0.06~4.7 ppm;<br>VX: 9.978×10^{-4} ~ 1.101×10^{-3};<br>HD: 0.61~4.9 ppm	PCA	Preliminary distinguish
						SVM	Accuracy > 90%
						KNN	Accuracy > 98%
						NNN	Accuracy ~ 100%

NA: Not available from the literature. DCP: 1,5-dichloropentane, a simulant of HD. DMMP: dimethyl methylphosphonate, a simulant of GB. ACN: acetonitrile, a simulant of AC. DCM: dichloromethane, a simulant of phosgene. DCNP: diethyl cyanophosphate, a simulant of GD. DFP: diisipropyl fluoride, , a simulant of the other kind of G-type organophosphorous nerve agents, i.e., Tabun (GA). LDA: linear discriminant analysis. PCA: principal component analysis. SVM: support vector machines. KNN: K-nearest neighbor algorithm. NNN: narrow neural networks.

S.I.2 Figures

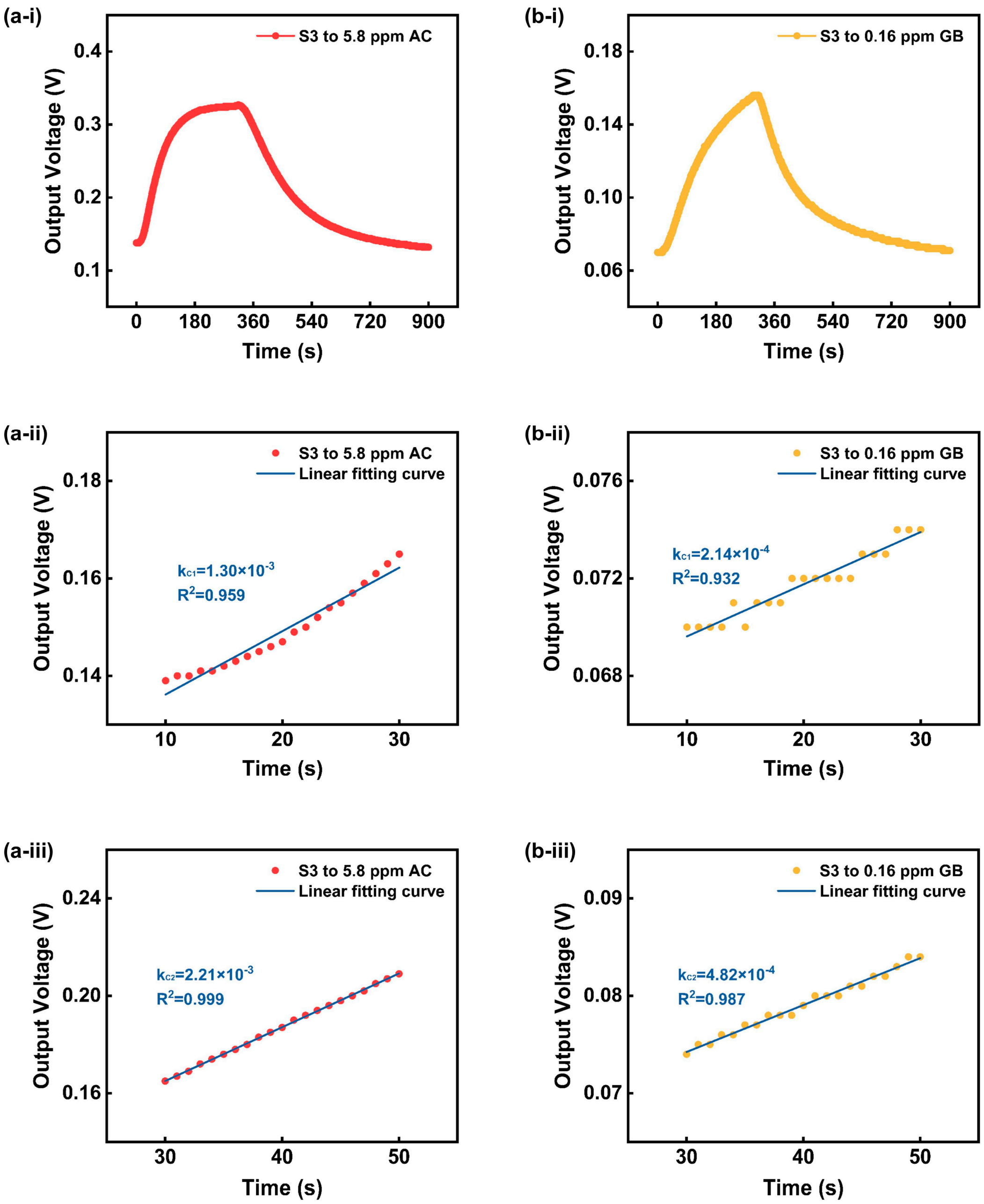

Figure S1 The dynamic response-recovery curve of S3 respond to **(a)** AC at 5.8 ppm, **(b)** GB at 0.16 ppm, and schematic diagram of extracting features from the kinetic characteristics of response curves (k_{C1}, k_{C2})

The slopes of the response curves of the output signals when the sensor was exposed to CWAs for 10 ~ 30 s and 30 ~ 50 s were obtained by linear fitting and denoted as k_{C1} and k_{C2}, respectively. Taking the response of S3 to 0.58 ppm AC and 0.16 ppm GB as examples, the results shown in Figure S1 are obtained. It can be seen that, the linear fitting R^2 corresponding

to the slope of the response curve for 10 ~ 30 s (k_{C1}) is greater than 0.9, and R^2 corresponding to the slope of the response curve for 30 ~ 50 s (k_{C2}) is greater than 0.98, indicating well fitting-effect. That is, k_{C1} and k_{C2} can represent the adsorption-reaction rate of the CWAs molecule on MOS surfaces in the initial response stage, to a certain extent.

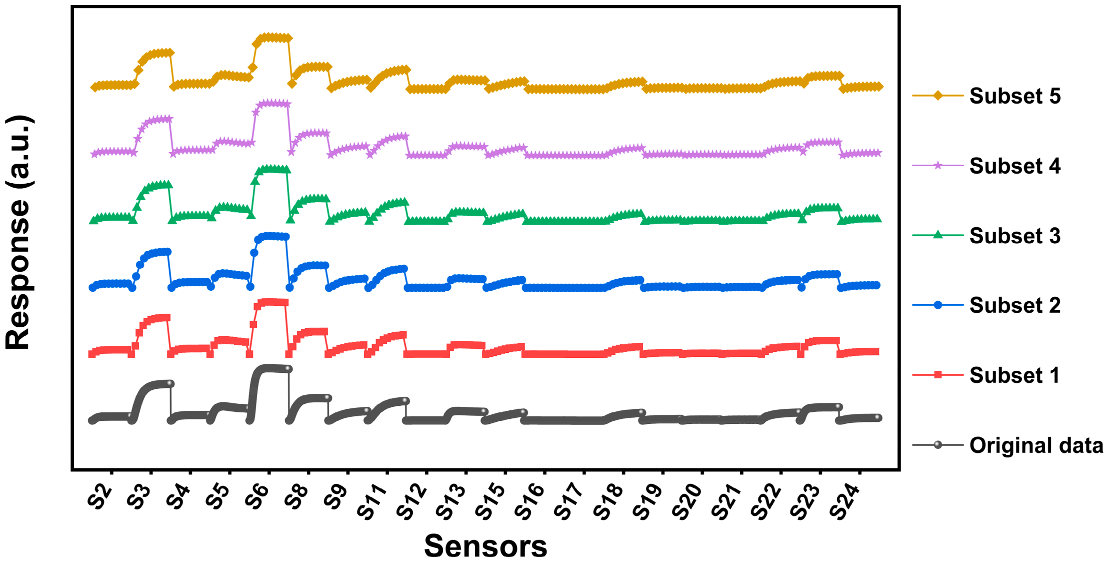

Figure S2 The pattern of a single response-recovery cycle of 20 sensos to at AC 16 ppm and the patterns of 5 subsets obtained by periodic interval sampling

Reference

[1] Pacsial-Ong, E.J. Chemical warfare agent detection a review of current trends and future perspective. *Front. Biosci.* 2013, S5, 516–543.

[2] Diauudin, F.N.; Rashid, J.I.A.; Knight, V.F.; Wan Yunus, W.M.Z.; Ong, K.K.; Kasim, N.A.M.; Abdul Halim, N.; Noor, S.A.M. A review of current advances in the detection of organophosphorus chemical warfare agents based biosensor approaches. *Sens. Bio-Sens. Res.* 2019, 26, 100305.

[3] Li, J.; Xie, G.; Dai, L.; Yang, M.; Su, Y. Rapid and highly selective dopamine sensing with CuInSe2-modified nanocomposite. *J. Compos. Sci.* 2025, 9, 123.

[4] Barreca, D.; Maccato, C.; Gasparotto, A. Metal oxide nanosystems as chemoresistive gas sensors for chemical warfare agents: A focused review. *Adv. Mater. Inter.* 2022, 9, 2102525.

[5] Yang, X.; Chen, S.; Zhang, H.; Huang, Z.; Liu, X.; Cheng, Z.; Li, T. Trace level analysis of nerve agent simulant DMMP with silicon nanowire FET sensor. *IEEE Sens. J.* 2020, 20, 12096–12101.

[6] Liu, X.; Zhang, H.; Huang, Z.; Yang, X.; Chen, S.; Wang, Y.; Li, T.; Cheng, Z. Silicon nanowire array sensor for highly sensitive and selective detection of nerve agent simulant vapor via surface hydroxyl groups. In Proceedings of the 2021 IEEE 16th

International Conference on Nano/Micro Engineered and Molecular Systems (NEMS), Xiamen, China, 25–29 April 2021; pp. 30–33.
[7] Yang, M.; He, J.; Hu, X.; Yan, C.; Cheng, Z.; Zhao, Y.; Zuo, G. Copper oxide nanoparticle sensors for hydrogen cyanide detection: Unprecedented selectivity and sensitivity. *Sens. Actuators B Chem.* 2011, 155, 692–698.
[8] Yang, M.; He, J.; Hu, X.; Yan, C.; Cheng, Z. CuO nanostructures as quartz crystal microbalance sensing layers for detection of trace hydrogen cyanide gas. *Environ. Sci. Technol.* 2011, 45, 6088–6094.
[9] Asri, M.I.A.; Hasan, M.N.; Fuaad, M.R.A.; Yunos, Y.M.; Ali, M.S.M. MEMS gas sensors: A review. *IEEE Sens. J.* 2021, 21, 18381–18397.
[10] Ollé, E.P.; Farré-Lladós, J.; Casals-Terré, J. Advancements in microfabricated gas sensors and microanalytical tools for the sensitive and selective detection of odors. *Sensors* 2020, 20, 5478.
[11] Sohn, J.R.; Park, H.D.; Lee, D.D. Acetonitrile sensing characteristics and infrared study of SnO2-based gas sensors. *Appl. Surf. Sci.* 2000, 161, 78–85.
[12] Lee, M.J.; Cheong, H.W.; Son, L.D.N.; Yoon, Y.S. Surface reaction mechanism of acetonitrile on doped SnO_2 sensor element and its response behavior. *Jpn. J. Appl. Phys.* 2008, 47, 2119–2121.
[13] Lee, W.S.; Lee, S.C.; Lee, S.J.; Lee, D.D.; Huh, J.S.; Jun, H.K.; Kim, J.C. The sensing behavior of SnO_2-based thick-film gas sensors at a low concentration of chemical agent simulants. *Sens. Actuators B Chem.* 2005, 108, 148–153.
[14] Yang, Z.; Zhang, Y.; Zhao, L.; Fei, T.; Liu, S.; Zhang, T. The synergistic effects of oxygen vacancy engineering and surface gold decoration on commercial SnO_2 for ppb-level DMMP sensing. *J. Colloid Interface Sci.* 2022, 608, 2703–2717.
[15] Sberveglieri, G.; Baratto, C.; Comini, E.; Faglia, G.; Ferroni, M.; Pardo, M.; Ponzoni, A.; Vomiero, A. Semiconducting tin oxide nanowires and thin films for chemical warfare agents detection. *Thin Solid. Film.* 2009, 517, 6156–6160.
[16] Tomchenko, A.A.; Harmer, G.P.; Marquis, B.T. Detection of chemical warfare agents using nanostructured metal oxide sensors. *Sens. Actuators B Chem.* 2005, 108, 41–55.
[17] Patil, L.A.; Bari, A.R.; Shinde, M.D.; Deo, V.; Kaushik, M.P. Detection of dimethyl methyl phosphonate—A simulant of sarin: The highly toxic chemical warfare—Using platinum activated nanocrystalline ZnO thick films. *Sens. Actuators B Chem.* 2012, 161, 372–380.
[18] Choi, N.J.; Kwak, J.H.; Lim, Y.T.; Bahn, T.H.; Yun, K.Y.; Kim, J.C.; Huh, J.S.; Lee, D.D. Classification of chemical warfare agents using thick film gas sensor array. *Sens. Actuators B Chem.* 2005, 108, 298–304.
[19] Olguín, C.; Laguarda Miró, N.; Pascual, L.; García Breijo, E.; Martínez Mañez, R.; Soto, J. An electronic nose for the detection of Sarin, Soman and Tabun mimics and interfering agents. *Sens. Actuators B Chem.* 2014, 202, 31–37.
